# Protein Folding Requires Crowd Control in a Simulated Cell

**DOI:** 10.1016/j.jmb.2010.01.074

**Published:** 2010-04-16

**Authors:** Benjamin R. Jefferys, Lawrence A. Kelley, Michael J.E. Sternberg

**Affiliations:** Division of Molecular Biosciences, Biochemistry Building, Imperial College London, South Kensington, London SW7 2AZ, UK

**Keywords:** TM, template modelling, macromolecular crowding, protein structure prediction, protein misfolding, protein aggregation, protein expression

## Abstract

Macromolecular crowding has a profound effect upon biochemical processes in the cell. We have computationally studied the effect of crowding upon protein folding for 12 small domains in a simulated cell using a coarse-grained protein model, which is based upon Langevin dynamics, designed to unify the often disjoint goals of protein folding simulation and structure prediction. The model can make predictions of native conformation with accuracy comparable with that of the best current template-free models. It is fast enough to enable a more extensive analysis of crowding than previously attempted, studying several proteins at many crowding levels and further random repetitions designed to more closely approximate the ensemble of conformations. We found that when crowding approaches 40% excluded volume, the maximum level found in the cell, proteins fold to fewer native-like states. Notably, when crowding is increased beyond this level, there is a sudden failure of protein folding: proteins fix upon a structure more quickly and become trapped in extended conformations. These results suggest that the ability of small protein domains to fold without the help of chaperones may be an important factor in limiting the degree of macromolecular crowding in the cell. Here, we discuss the possible implications regarding the relationship between protein expression level, protein size, chaperone activity and aggregation.

## Introduction

It is widely acknowledged that there are two fundamental questions in modelling protein folding:^1^

What is the biologically active conformation of a protein sequence?
How does a protein find that state in the cell?

These two problems have generally been approached using radically different methods. The most successful methods for predicting the biologically active state are through finding a homologue of the sequence to another sequence with known structure or through assembly of fragments of structure when such homology cannot be detected. These are valuable tools for suggesting the structure of a protein sequence where no experimental structure is available, and they consistently perform well at CASP (Critical Assessment of techniques for protein Structure Prediction) experiments.^2–7^

However, such methods provide little, if any, insight into the principles underlying the search for that state: the speed and reliability of folding, the stability and flexibility of the final conformation, the effect of conditions in the cell, misfolding and aggregation and error-correcting processes. Molecular dynamics is used for investigating these elements of protein folding.^8^ Although progress is being made toward predicting protein structure through molecular dynamics,^9^ such methods are not yet among the most successful predictive tools and the large computational resources required make their application to large-scale analysis impractical.

Simplifications of molecular dynamics that reduce the number of particles modelled, simplify the force field and use time-saving heuristics in the search method^1,10^ enable larger-scale analyses of protein folding. Ultimately, the goal is to study the mechanism of protein folding and obtain the biologically active conformation with a unified model for protein structure, which predicts the native state through accurate modelling of the folding process. We present here a protein model developed for this purpose and demonstrate its effectiveness in both protein structure prediction and the study of the protein folding process. The structural model reduces the backbone to a series of particles representing C^α^ atoms and particles representing the centroid of side-chain atoms.^11^ The folding model is a simplified iterative solution of Newtonian equations of motion based upon Langevin dynamics, with linear elastic springs modelling the majority of effects in the force field. Solution of this equation aims to simulate protein folding over time through a putative pathway. The Langevin equation includes a term for random bombardment by implicit solvent; therefore, the generated folding pathway is dependent upon the seed for a random number generator and multiple pathways must be produced to assess the ensemble of conformations. Such a model can be used to investigate many aspects of protein folding, including the effect of conditions in the cell, such as macromolecular crowding.

The cell is a crowded and chaotic environment: it is estimated that between 10% and 40% of the cell's volume is occupied by macromolecules.^12,13^ However, complex and intricate biochemical processes must be performed reliably in this environment. A minimum concentration of molecules in the cell is necessary for interacting partners to associate. There is also a maximum concentration beyond which normal cellular function would be prevented due to restriction of molecular motion. Evidence suggests that crowding enhances protein stability, protein association and chaperonin action but that it also increases protein aggregation and lowers diffusion rates.^12^ The macromolecular concentration observed in cells may have been selected to balance these factors, according to requirements in different cellular localities.^14,15^ In this study, we investigated the role of protein folding in this balance.

Previous work suggests that macromolecular crowding has a complex effect upon protein folding.^12,16^ Computational models and experimental work show that, at a crowding level similar to that of the cell as a whole, crowding can make proteins fold more rapidly and stabilise the native state at secondary and tertiary levels.^17–22^ This effect is interpreted as being due to the reduction of conformation space by exclusion of extended conformations.

Computational studies of the effect of crowding upon protein folding commonly use a Gō-like term^23^ in the energy function for the protein, which favours interactions known to occur in the native conformation.^19,21,22,24^ Clearly, such terms disqualify these methods as tools for predicting the final conformation. As a consequence of using a Gō-like potential, terms may be omitted for non-native but favourable interactions that might be encountered on the way to the native state—interactions that might be important in finding the native state or in creating a non-native local energy minimum.^25^ If the effect of crowding were to trap a protein in such a minimum, then this effect would not be observed with a Gō-like potential.

Simulations making use of molecular dynamics do not have this disadvantage. However, due to computational limitations, they often study only the change in stability of proteins in their native state when subjected to crowding,^8,19^ and where proteins have been refolded from a partially denatured state, only very small proteins were studied.^19^ A study using molecular dynamics has shown that crowding may destabilise the native state of the protein by forcing the solvent into a more ordered state, reducing the entropic advantage of the native over other conformations.^8^ Native stability and refolding rates are important aspects of the protein folding problem. However, in the cell, proteins are synthesized slowly into a crowded environment. While computational analysis has found that co-translational folding appears to have a small effect on protein folding rate and has only weakly imprinted motifs in structures that might be expected if it did,^26–29^ experimental observations are more mixed.^30,31^ Slow synthesis may allow proteins to find a compact state more easily than from a denatured state, an effect that may be even more important in a crowded environment. Additionally, computational and experimental constraints have limited previous studies to one or two structures and few crowding levels. This raises the possibility that effects observed are specific to the chosen conditions.

To address these issues, we have studied the effect of crowding using a protein model that does not include knowledge of long-range interactions, can predict protein tertiary structure with accuracy comparable with that of the best current template-free methods, includes a simulation of the folding pathway during slow synthesis of a protein by a large, heavy ribosome and is fast enough to allow study of many proteins at several crowding levels. The aim of this work was to find the upper limit of crowding in which proteins can successfully fold and compare this with the level of crowding observed in the cell.

## Results

### Model verification through structure prediction

**Fig. 1:**
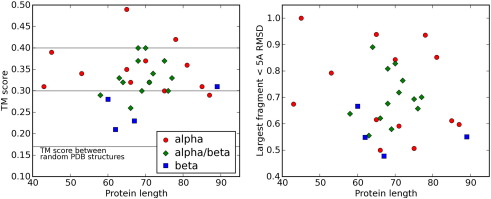
Structure prediction results. TM scores for the proteins are shown on the left. The shape and colour indicate broad protein class: red circles are all-helical, blue squares are all-strand and green diamonds are mixed. Twenty-four targets were predicted with structures better than 0.3 in TM score, and four of those were predicted with structures better than 0.4 in TM score. For comparison, we plotted the largest proportion of the protein that can be aligned to the native at less than 5 Å RMSD on the right. The point shape and colour are as those for the graph on the left.

**Fig. 2:**
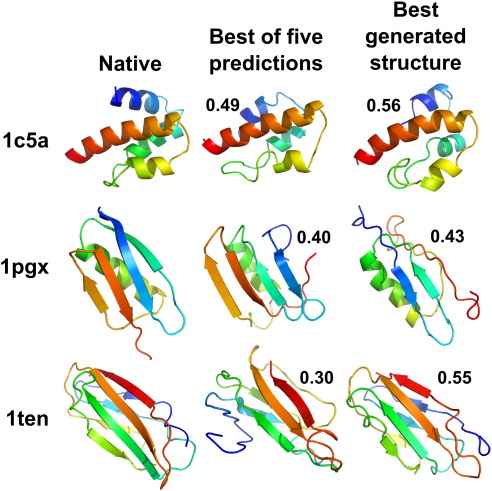
Best of five predictions and the best structure (by TM score) generated by the model during structure prediction for an all-helical protein, a mixed protein and an all-sheet protein. Proteins are shown coloured from red at the C-terminus to blue at the N-terminus, with arrows representing strands and ribbons representing helices. The best TM score structure in the right-hand column shows the best conformation, as compared with the known native structure, out of several thousands of structures produced by 150 separate folding trajectories and therefore does *not* represent a prediction but rather the theoretical best prediction that could be made by *poing*. Images were generated using PyMOL.

We tested our model by making predictions of the tertiary structure for a set of 30 small protein domains (listed in Supplementary Material) that were chosen for the quality of experimental structure, size and fold class. The structure prediction protocol is described in Materials and Methods. Modelling is based upon the predicted secondary structure using psipred.^32^ Five structure predictions are made for each protein, and the best TM (template modelling) score^33^ and largest fraction of residues alignable to within 5 Å to the native are given in Fig. 1. The TM score is a measure of structural similarity that is more sensitive to the similarity of protein fragments than the more commonly used global RMSD (root-mean-square deviation). The mean TM score between randomly chosen Protein Data Bank structures is 0.17, and a TM score exceeding 0.3 indicates a roughly native-like topology. It is unusual to exceed a TM score of 0.4 in template-free modelling. The best of the five predictions for 24 of the proteins has a TM score higher than 0.3, and for 4 of those proteins, the score is higher than 0.4. Predictions and the best structures generated for 3 of the proteins are illustrated in Fig. 2.

These results mean this method is comparable with the best template-free structure prediction methods.^34^ Each prediction is made from simulations using at most 40 CPU hours, a tiny fraction of the time that is used by many other successful template-free predictive methods. It is this short processing time that has enabled this work studying protein folding in detail under hundreds of experimental conditions.

Our model iteratively generates putative folding pathways using basic physical principles. While we have not analysed how these pathways compare with experimental data that might suggest the natural folding process for a protein, the good results from predicting tertiary structure using conformations sampled from these pathways suggest that they are reasonable. Although the amount of good-quality data regarding the mechanism of folding is growing, it is still small compared with the huge and reliable database of protein structures, and therefore those data are used as a metric to assess the performance of our model.

### Effect of crowding upon protein folding

**Fig. 3:**
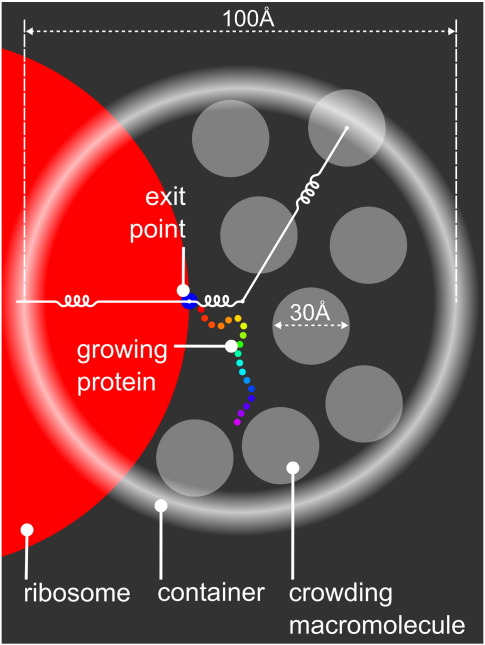
Illustration of model for synthesizing protein into a crowded container. The white curly lines represent springs that hold together the ribosome, ribosome exit point and container and keep particles inside the container. The ribosome is the large red circle (only partially visible), the ribosome synthesis exit point is the small blue circle on the ribosome's surface, the crowding macromolecules are in translucent gray and the protein is shown with colours going from red at the C-terminus to purple at the N-terminus.

**Fig. 4:**
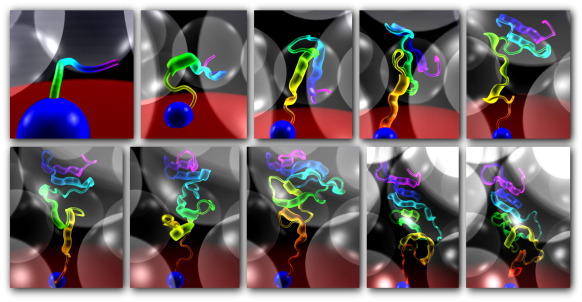
A series of images showing the synthesis of a protein into a cell with 41% volume excluded by macromolecules. Each image is approximately 10,000 time steps apart. The protein is shown in cartoon form, with ribbons showing the assigned secondary structure. Colour scheme is as that for Fig. 3, with translucent crowding macromolecules. Note that the protein is becoming squeezed between the crowding macromolecules as it is synthesized. Images were generated by *poing*, the protein model used for this work.

**Fig. 5:**
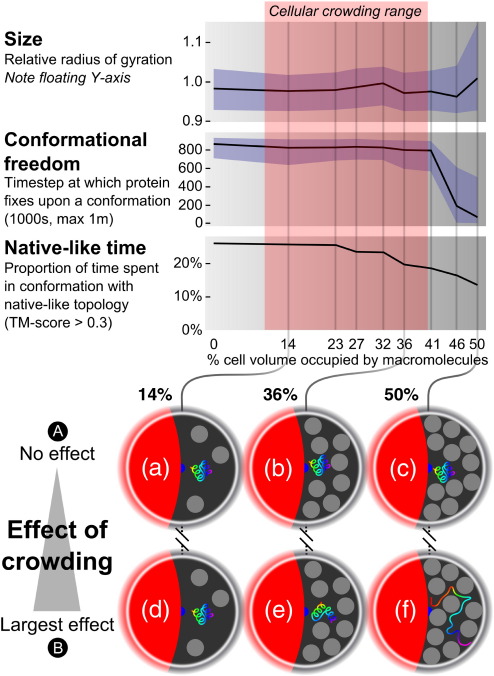
Effects of crowding upon conformation size, conformational freedom and native-like time. *N* = 1.2 million for each crowding level for all graphs, although structures produced during simulation of a single folding trajectory are not strictly independent, and 1200 folding trajectories were simulated. The illustrations at the bottom show how crowding changes the space remaining for the protein to fold. Colour scheme is as that for Fig. 3. All elements have proportions designed to show the relative excluded volume resulting from crowding as relative excluded area in two dimensions—for example, at 50% crowding, 50% of the area within the container not occupied by the ribosome is occupied by macromolecules. (Row A) These illustrate the space that remains for a successfully folded protein. Space is limited at 50% (c), but there is still space for more proteins in the three-dimensional case. (Row B) These illustrate the largest effect of crowding upon folding. At 14% (d), there is no measurable effect on the folded protein. At 36% (e), proteins are starting to get trapped in non-native but compact conformations. At 50% (f), proteins are becoming trapped in extended conformations.

The effect of crowding upon protein folding was investigated for a subset of the proteins used to test the model through structure prediction. Of the 30 proteins, 12 (listed in Supplementary Material) for which the model performs well were chosen, producing structures with a TM score higher than 0.3 for at least 10% of the total simulation during 100 syntheses of the protein. These proteins were then studied under a range of crowding conditions. We assume that the primary crowding agent at the time of protein folding is another previously synthesized protein of size similar to that which is being folded. Other crowding macromolecules are likely to be present in the cell; however, the effect of heterogeneous crowding as compared with homogeneous crowding is beyond the scope of this work and left for future investigation. It is observed from experimental work that the specific physicochemical properties of the crowding macromolecules are not especially important, rather it is the volume of solvent excluded by the molecules that has the greatest influence upon protein folding.^16,17,35^ Therefore, crowding was simulated by spheres with radius and mass designed to simulate the volume excluded by a small domain. Modelling a protein-like crowding macromolecule at this coarse level of detail makes this study computationally feasible: a more detailed model would slow simulations by orders of magnitude. Since the primary concern at this stage is folding rather than structure prediction, we used secondary structure assigned from the known native using Stride.^36^ Each of the 12 proteins was synthesized 100 times into a cell with a number of crowding macromolecules according to the desired crowding level. After synthesis, simulation continued for 1 million iterations and conformations were sampled at 1000 iteration intervals, this being sufficient to allow local rearrangement of secondary structure elements. In summary, 1000 conformation samples were taken from each of 100 folding simulations, producing 100,000 protein conformations for each protein at each crowding level. Since 12 proteins were selected for this analysis, 1.2 million conformations in total were produced to assess the effect of a given level of crowding. The simulation is illustrated in Fig. 3, and a series of images taken at regular iteration intervals from a simulation synthesizing a protein into a cell with the maximum experimentally observed crowding level is shown in Fig. 4. To determine how the changing environment affects different aspects of folding, we made measurements of conformation size, conformational freedom and time spent in a native-like state as volume excluded by crowding macromolecules increases. Results are summarised in Fig. 5.

#### Effect of crowding upon conformation size

Crowding may result in more compact proteins through exclusion of extended conformations or in less compact proteins through trapping in extended conformations. In order to investigate this, we measured conformation size using radius of gyration relative to the radius of gyration of the native state. The median (black line) and middle 50th (blue region) percentiles of the distribution of these radii are given in the top graph in Fig. 5. Conformations are generally compact up to 45% excluded volume. At 50% excluded volume, many conformations are less compact. Visual examination of the most extended conformations shows that they are trapped in narrow corridors of solvent between the crowding particles, as illustrated in (f) in Fig. 5.

#### Effect of crowding upon conformational freedom

Crowding may change the conformational freedom of proteins—they may be stabilised or destabilised by collisions with macromolecules. We assess the time step of each simulation at which a protein finds a conformation that does not change significantly for the rest of the simulation. Conformations are defined as being similar if the mean difference in pairwise distances between corresponding amino acids is less than 4 Å. The reported time step for a given simulation is that at which a conformation that is similar to all conformations after it until the end of the simulation is found. The median (black line) and middle 50th (blue region) percentiles of this number are shown in the middle graph in Fig. 5. Freedom remains fairly constant up to 30% excluded volume, with most of the simulated proteins free to explore conformation space until around 800,000 iterations (out of 1 million). Above 40% excluded volume, conformational freedom decreases sharply: most simulated proteins fix upon a structure before 200,000 iterations. Combining this with the observations on conformation size, at 50% excluded volume, it would seem that proteins are becoming trapped in an extended state. At slightly below this level, proteins are generally trapped in a compact state.

#### Effect of crowding upon native-like time

Crowding may increase or decrease the similarity of the protein to the native state during the simulation. We assess this effect to be measuring native-like time, which we define as the percentage of sampled conformations that are higher than 0.3 in TM score to the native state. The percentage across all proteins is shown in the bottom graph of Fig. 5. Native-like time remains fairly constant up to 25% excluded volume, with over 20% of conformations being above 0.3 in TM score. There is an initial slight drop up to 30% crowding, which cannot be attributed to more extended conformations or becoming trapped in a conformation early. Above this level, native-like time reduces rapidly, until at 50% excluded volume, just over 10% of conformations have a TM score above 0.3. The large drop in native-like time at 35% coincides with a slight reduction in conformational freedom, although conformation size remains fairly constant. Some proteins become stabilised in compact but non-native conformations by the presence of crowding particles, illustrated in (e) in Fig. 5. This effect becomes more acute at 45% excluded volume, when conformational freedom drops sharply. Finally, when crowding is so great the proteins cannot find a compact conformation, proteins very quickly become trapped in extended states that are clearly less likely to be similar to the native [illustrated in (f) in Fig. 5].

In summary, there is little effect on the measured features up to 20% excluded volume. At around 25%, native-like time begins to reduce, although not due to being fixed early or trapped in extended conformations. Above 40% crowding, proteins are becoming stuck in structures earlier in the simulation, and around 15 of the structures are in native-like topologies, compared with 14 with no crowding. At 50% crowding, proteins are stabilised in extended conformations early in the simulation in narrow gaps between macromolecules.

## Discussion

We have presented a protein structure model designed to predict tertiary structure without the use of a template or detailed knowledge of the native state. The model demonstrates accuracy comparable with that of the most successful methods, such as fragment folding, with the added benefit that it is fast and models a putative folding pathway. Using this model, we have tested the effect of macromolecular crowding upon the folding of 12 small protein domains synthesized into a simulated cell.

Crowding macromolecules in the simulated cell were simulated using same-sized spheres up to an excluded volume of 50%. As crowding is increased to experimentally observed levels (20% to 40%), a slow decrease in native-like time, due to the stabilisation of non-native but compact states, is observed. However, when the crowding level surpasses the maximum observed experimentally, conformation size and conformational freedom change sharply and native-like time drops substantially. This represents a level of crowding beyond that observed experimentally, yet still far less than the maximum achievable by close packing spheres (74%),^37^ leaving more than sufficient space for a protein to fold—especially given that the modelled crowding macromolecules can move to accommodate the growing protein.

The results presented here have shown the effect of folding upon the protein folding pathway at a coarse level. The general finding that proteins fix upon a protein structure very much earlier in an overcrowded cell, relative to uncrowded conditions, is not intended to be a prediction of the level of conformational frustration but is indicative that this is a possible cause of the reduction in successful protein folding at extreme crowding levels. The folding pathways produced by the iterative process in our model have not been verified in detail; therefore, a study of the effect of crowding upon such detail would be premature at this stage. However, future work based upon such verification may reveal more about the specific cause of the effect we have observed. In particular, the relative influence of two-state *versus* cooperative folding processes under crowded conditions, as compared with proteins in pure water, may be important.

The importance of crowding to cellular processes is clear. High levels of crowding are known to increase protein aggregation, which can lead to cell death, and substantial evidence that prevention of aggregation is a key factor in protein evolution exists.^38^ It is clear that reliable protein folding is critical to the survival of the cell. Hence, factors that detrimentally affect this process are expected to be under strong selective pressure. This work suggests that crowding above 40% excluded volume severely hinders folding. The agreement between this maximum value of crowding tolerated in our simulation and that found experimentally suggests that the ability to fold proteins reliably in the cell may be an important evolutionary constraint upon the level of macromolecule concentration.

Macromolecular crowding is influenced by two key factors: protein size and expression level. The well-established inverse relationship between these two factors^39–41^ is generally interpreted as being due to evolutionary minimization of transcriptional and translational costs. However, the results of this work suggest that this relationship may also be in part due to the balancing of size and expression to maintain a crowding level that permits folding. This suggestion is supported by the strong anti-correlation between expression level and propensity to aggregate^42^ and by evidence that larger proteins are more prone to aggregation.^43^

Crowding may have been controlled during evolution to allow the folding of small domains without recourse to error-correcting mechanisms, such as chaperones and directed proteolysis. Chaperones associate preferentially with larger domains, generally above 200–300 amino acids,^44–46^ suggesting that error-correcting mechanisms such as chaperones may have evolved in part to enable crowding to increase beyond the level tolerated by larger domains.

This work demonstrates a theoretical model that unifies the often disjoint goals of protein structure prediction and modelling folding dynamics, using it to study the effect of crowding to a level of detail that would be difficult to achieve experimentally. Further development of such a model can enable study of protein flexibility, complex formation, sequence design for synthetic biology and disease-causing misfolding and aggregation.

## Materials and Methods

### Protein structure and force field

The model, known as *poing*, reduces a protein structure to C^α^ points plus side-chain centroids.^11^ It models structures through iterative prediction of a folding pathway that enforces a number of heuristic constraints representing effects important in protein folding. *poing* uses the Langevin equation^47^ for the motion of a particle in the system:

(1) $$\vec{a}=\frac{\vec{F}-\gamma \vec{v}+\vec{R}}{m}$$

where *a⃗* is the acceleration of the particle, *F⃗* is the force field at the coordinates of the particle (the sum of all forces acting on the particle), γ is a drag factor due to motion through the implicit solvent, *v⃗* is the current velocity of the particle, *R⃗* is a random force vector designed to model the effect of kicks from the implicit solvent and *m* is the mass of the particle. This equation is solved iteratively. The force field consists of a set of pairwise force functions that act upon the particles:

(2) $$\vec{F}={\vec{F}}_{\mathrm{cov}}+{\vec{F}}_{\text{bb}}+{\vec{F}}_{\text{sc}}+{\vec{F}}_{\text{vdw}}+{\vec{F}}_{\text{hb}}$$

*F⃗*_cov_ represents stiff springs linking backbone and side-chain particles that are directly covalently bonded. *F⃗*_bb_ represents springs between backbone particles with sequence separations of 2 or 3, with a range of equilibrium lengths based on secondary structure assigned to the relevant amino acids, either from secondary structure prediction (when used as a predictive tool) or from knowledge of the native (when used to simulate folding pathways). *F⃗*_sc_ represents springs linking side-chain particles to neighbouring backbone particles, controlling the orientation of side chains relative to the backbone. *F⃗*_vdw_ is a repulsive force derived from the probability that atoms in an all-atom model of those particles at a given distance apart would clash sterically, based upon analysis of side-chain and backbone conformations in the Protein Data Bank.^48^ *F⃗*_hb_ is an attractive force between backbone particles designed to bring virtual backbone hydrogen bonding O and H atoms into closer proximity, if they already come within a distance threshold.

### Hybrid implicit–explicit solvent with hydrophobic effect

The standard Langevin has an implicit solvent model, with drag (−γ*v⃗*) and kick (*R⃗*) terms in the main equation. We have enhanced this by ensuring that drag and kicks only act upon parts of a particle exposed to solvent, with solvent-accessible surfaces modelled by spheres around each particle of a radius dependent upon the side-chain type. This ensures that the internal parts of a protein are not subject to solvent effects, a key advantage of modelling an explicit solvent. The solvent-accessible radii used have been optimised to maximise the difference in accessible area between known native and a set of non-native states for a small test set of proteins, to destabilise non-native states.

The solvent kick model is modified from the normal Langevin to enable this process. At each time step, a kick is initiated upon a particle with some probability per Å^2^ accessible surface area. All kicks are of the same velocity. If the kick does not come from a direction that is blocked by other particles, it is added to the acceleration for that particle. The probability of kicks is increased for hydrophobic side-chain particles. This results in preferential burial of hydrophobic residues away from solvent and therefore the hydrophobic collapse of a protein molecule. This is the only effect of the hydrophobicity of a side chain in the model.

### Structure prediction

Given a sequence of amino acids, the initial step is to predict the secondary structure using psipred,^32^ a neural network-based secondary structure prediction tool that takes as input position-specific scoring matrices derived from homologous sequences found with PSI-BLAST. The accuracy of this method generally reduces where no homologue is found. A simple three-state output (helix, extended/strand and coil) is used to assign secondary structure to the amino acids in the *poing* model. The protein is then modelled in *poing*, for (6000*l* + 400,000) iterations, where *l* is the number of amino acids in the protein. Forty structures are sampled at equal intervals from iteration 6000*l* to the end of the simulation. As a computational shortcut to a compact structure, the protein is slowly synthesized by adding an amino acid to the C-terminus of the protein every set of 1000 iterations and tethering this growing end to the edge of a large heavy sphere representing a ribosome. This process is repeated 150 times with different random seeds for the generation of kicks from solvent in the Langevin equation, producing many folding trajectories. This produces the final pool of structures from which predictions are made.

Determination of structure predictions from generated conformations is performed in three stages. First, a mean of the contact maps of the backbone particles of the pool of structures is generated. The contact maps are calculated based upon backbone traces smoothed with a window of nine amino acids. The contact maps disregard contacts between amino acids within the same secondary structure unit. Different distance cutoffs are used depending on the secondary structure: 8 Å between amino acids that are both assigned as strand and 11 Å in all other cases. This reflects the fact that the backbones of hydrogen-bonded strands are closer than other secondary structure elements. All 6000 structures are sorted according to their similarity to this mean contact map—the contact map similarity score penalises a lack of contact where there should be one and rewards the presence of a contact where there should be one.

The top 20 structures selected by similarity to a consensus contact map are scored using ProQ,^49^ a neural net-based structure scoring program trained to predict the MaxSub score of a protein as compared with its (unknown) native state. If any of the top 5 structures picked by ProQ are very similar to one another, the one with the lowest score is eliminated and the next structure down is brought in to the top 5. Two proteins are judged to be very similar if more than 90% of the residues can be aligned to less than 7 Å RMSD. This process is repeated until the top 5 represents a range of the best structures produced by *poing*, or there are no more structures.

### Model for protein synthesis into a crowded cell

This is illustrated in Fig. 3. The model consists of a simulated protein (described above) and crowding macromolecule spheres inside a containing sphere and a heavy ribosome with a synthesis exit point located on its surface, with the exit point tethered to the centre of the containing sphere. The ribosome, crowding macromolecules and containing sphere all move iteratively under forces applied through the Langevin equation, including the solvent model. Details of these elements are given below.

In *poing*, the ribosome is modelled with a heavy sphere of radius 100 Å. All simulated particles are excluded from this sphere. The mass is set to make the ribosome an essentially immovable object. The protein emerges from an exit tunnel on the surface of a ribosome, to which it is tethered: this is modelled by a particle attached to the surface of the ribosome. This particle has the same mass as the ribosome in order to approximate the moment of inertia of the ribosome as a whole. The backbone particle at the growing end of a protein is attached to this particle. This set of strong springs and massive particles presents a major constraint to the protein's freedom, which is designed to approximate the effect of the ribosome on protein folding.

Protein synthesis is modelled by periodically adding backbone and side-chain particles to the C-terminus chain at the position of the exit tunnel. The new backbone point is tethered to the exit point, and the old backbone point to which it is attached is no longer tethered (however, its location is still restricted by its backbone link to the new backbone particle). We used a synthesis rate of one amino acid per 1000 time steps. This is an artificially fast synthesis rate, relative to the rate of protein collapse in our model; however, short tests have found that slower synthesis rates do not alter our observations. This relatively fast rate was chosen as a computational optimisation.

Macromolecular crowding is designed to simulate the crowding of a protein by other proteins of a similar size. Previous work^17^ has suggested that the specific physicochemical properties of the crowding macromolecules are not especially important and that the principal effect is volume exclusion. It would be computationally prohibitive and unnecessary to crowd a protein with other complete protein models if there is no requirement to simulate specific interactions. Therefore, in *poing*, a crowding macromolecule is reduced to a large, heavy sphere. The macromolecules crowding a protein are assumed to be of size similar to the protein being crowded. The set of small domains used in this study is between 43 and 90 amino acids long. All particles in the protein model have the same mass, and each amino acid has two particles associated with it. This is simplified to a single crowding particle that models a 75-amino-acid protein, of diameter 30 Å (the approximate size of a 75-amino-acid protein) and with mass 150 times that of a single particle. The entire system of particles (excluding the ribosome) is contained within a sphere of diameter 100 Å.

The ribosome exit point is tied to the centre of the crowding containment sphere with a spring, ensuring that the protein is synthesized into the centre of the crowding area. This ensures that any observed effects upon folding from crowding are due to the crowding rather than boundary-specific effects that might be observed if the protein is synthesized at the boundary.
