# Supplementary material for: Protein Folding Requires Crowd Control in a Simulated Cell
Source: J Mol Biol. 2010 Apr 16;397(5):1329–38. doi: 10.1016/j.jmb.2010.01.074 (PMC2891488; doi:10.1016/j.jmb.2010.01.074)
Supplement: Supplementary file 1 — Supplementary Material [file mmc1.pdf]

Supplementary material

Proteins used for prediction and crowding studies

PDB code	SS	Length	Description (from PDB entry)	Used in crowding study?
1a6s	α	87	Bioactive retroviral M domain from *Rous sarcoma* virus	No
1a8o	α	66	C-terminal dimerization domain of the HIV-1 capsid protein	Yes
1aa3	$\alpha\beta$	63	C-terminal domain of *E. coli* RecA protein	No
1ail	α	70	RNA-binding domain of the influenza virus NS1 protein	Yes
1aiw	β	62	Cellulose-binding domain of the endoglucanase Z secreted by *Erwinia chrysanthemi*	No
1c5a	α	65	Porcine C5ades*Arg from 1H	Yes
1csp	β	67	Universal nucleic acid-binding domain from *B. subtilis* major cold-shock protein	No
1ctf	$\alpha\beta$	68	C-terminal domain of the ribosomal protein L7/L12 from *E. coli*	Yes
1dv0a	α	45	C-terminal UBA domain of the human homologue of RAD23A	No
1fwp	$\alpha\beta$	69	CheY-binding domain of the chemotaxis kinase CheA	No
1gab	α	53	Albumin-binding GA module	Yes
1hyp	α	75	Hydrophobic protein from soybean	Yes
1ig5a	$\alpha\beta$	75	Bovine calbindin D9K binding Mg2+	Yes
1lea	$\alpha\beta$	72	LexA repressor DNA binding domain	Yes
1msi	$\alpha\beta$	66	Structure of antifreeze glycoprotein QAE	No
1ngr	α	85	Death domain of the p75 neurotrophin receptor	No
1nkl	α	78	NK-lysin from pig	Yes
1nre	α	81	N-terminal domain of alpha2-macroglobulin receptor-associated protein	Yes
1orc	$\alpha\beta$	64	Cro repressor insertion mutatnt K56-[DGEVK]	No
1pgx	$\alpha\beta$	70	B2 immunoglobulin-binding domain of streptococcal protein G	No
1pou	α	71	Oct-1 POU-specific domain	Yes
1res	α	43	DNA binding domain of gamma delta resolvase	No
1stu	$\alpha\beta$	68	dsRNA binding domain from *Drosophila* staufen protein	No
1ten	β	90	Fibronectin type III domain from tenascin	No
1vcc	$\alpha\beta$	77	N-terminal fragment of vaccinia virus DNA topoisomerase I	No
1vif	β	60	Dihydrofolate reductase from trimethoprim-resistant bacteria	No
1vig	$\alpha\beta$	71	Vigilin KH domain	No
2ezh	α	65	I gamma subdomain of the Mu end DNA-binding domain of phage Mu transposase	Yes
2fow	$\alpha\beta$	76	RNA binding domain of ribosomal protein L11	No
5pti	$\alpha\beta$	58	Bovine pancreatic trypsin inhibitor	No
